# Human muscle stem cells are refractory to aging

**DOI:** 10.1111/acel.13411

**Published:** 2021-06-05

**Authors:** James S. Novak, Davi A. G. Mázala, Marie Nearing, Ravi Hindupur, Prech Uapinyoying, Nayab F. Habib, Tessa Dickson, Olga B. Ioffe, Brent T. Harris, Marie N. Fidelia‐Lambert, Christopher T. Rossi, D. Ashely Hill, Kathryn R. Wagner, Eric P. Hoffman, Terence A. Partridge

**Affiliations:** ^1^ Center for Genetic Medicine Research, Children's Research Institute Children's National Hospital Washington DC USA; ^2^ Department of Genomics and Precision Medicine The George Washington University School of Medicine and Health Sciences Washington DC USA; ^3^ Department of Pediatrics The George Washington University School of Medicine and Health Sciences Washington DC USA; ^4^ Department of Kinesiology, College of Health Professions Towson University Towson MD USA; ^5^ Department of Medicine University of Alabama at Birmingham Birmingham AL USA; ^6^ Neuromuscular and Neurogenetic Disorders of Childhood Section, National Institute of Neurological Disorders and Stroke National Institutes of Health Bethesda MD USA; ^7^ Department of Pathology University of Maryland School of Medicine Baltimore MD USA; ^8^ Department of Neurology and Pathology Georgetown University Medical Center Washington DC USA; ^9^ Department of Pathology Howard University Hospital Washington DC USA; ^10^ Department of Pathology and Laboratory Medicine Children's National Hospital Washington DC USA; ^11^ The Hugo W. Moser Research Institute Kennedy Krieger Institute Baltimore MD USA; ^12^ Departments of Neurology and Neuroscience Johns Hopkins University School of Medicine Baltimore MD USA; ^13^ Department of Pharmaceutical Sciences, School of Pharmacy and Pharmaceutical Sciences Binghamton University Binghamton NY USA

**Keywords:** aging, human satellite cells, muscle regeneration, myogenic capacity, sarcopenia

## Abstract

Age‐related loss of muscle mass and strength is widely attributed to limitation in the capacity of muscle resident satellite cells to perform their myogenic function. This idea contains two notions that have not been comprehensively evaluated by experiment. First, it entails the idea that we damage and lose substantial amounts of muscle in the course of our normal daily activities. Second, it suggests that mechanisms of muscle repair are in some way exhausted, thus limiting muscle regeneration. A third potential option is that the aged environment becomes inimical to the conduct of muscle regeneration. In the present study, we used our established model of human muscle xenografting to test whether muscle samples taken from cadavers, of a range of ages, maintained their myogenic potential after being transplanted into immunodeficient mice. We find no measurable difference in regeneration across the range of ages investigated up to 78 years of age. Moreover, we report that satellite cells maintained their myogenic capacity even when muscles were grafted 11 days postmortem in our model. We conclude that the loss of muscle mass with increasing age is not attributable to any intrinsic loss of myogenicity and is most likely a reflection of progressive and detrimental changes in the muscle microenvironment such as to disfavor the myogenic function of these cells.

## Graphical abstract

Sarcopenia, or age‐related muscle atrophy, leads to significant impairments in muscle function and strength in the elderly. We developed a model of human muscle xenografting to assess myogenic capacity of human cadaver muscles across a range of ages. We find no difference in the regenerative potential of human muscle stem cells based on age and conclude that sarcopenia is most likely a reflection of detrimental changes in the muscle micro‐environment that disfavor myogenic capacity with advanced age.

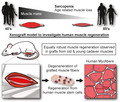

## 1 INTRODUCTION

Sarcopenia, age‐related loss of skeletal muscle, is the most prevalent type of muscle atrophy in humans leading to significant impairments of function and strength (Rosenberg, 1997). In humans, sarcopenia typically begins to intrude around the 4th decade of life at a rate of roughly 1% muscle loss annually amounting, in some individuals, to a cumulative loss of 30%–50% skeletal muscle mass by 80 years of age (Lexell et al., 1988). This process is heavily influenced by a panoply of factors stemming from the inflammatory, fibrogenic and adipogenic cells that constitute the muscle interstitial microenvironment (Blau et al., 2015; Brown et al., 2020). With increasing age, the propagation of pro‐inflammatory cytokine signaling and infiltration of fibrotic and adipogenic tissue within the muscle, together, have been shown to exert detrimental impacts on muscle satellite cell (MuSC) quiescence and regenerative capacity (Blau et al., 2015; Bouche et al., 2014; Brack et al., 2007; Brown et al., 2020; Chakkalakal et al., 2012; Wang et al., 2017). Sarcopenia is intimately associated with many age‐related pathologies, including frailty, Alzheimer's disease, type 2 diabetes, and others (Foster et al., 2011; Santilli et al., 2014; Tolea & Galvin, 2015).

Although a number of investigations of the basis of sarcopenia have implicated defective motor inervation as an important factor (Butikofer et al., 2011; Faulkner et al., 2007; Kulakowski et al., 2011; Li et al., 2011), attenuation of myogenic competence is also attributed a major role. Some investigations have demonstrated a major extrinsic component to myogenic competence in aged individuals (Bernet et al., 2014; Carlson & Faulkner, 1989; Collins et al., 2007; Conboy et al., 2005; Cosgrove et al., 2014; Sousa‐Victor et al., 2014), but other studies have implicated intrinsic defects in the behavior of aged MuSCs as playing an important part in the failure to maintain muscle mass, MuSC senescence, and regenerative capacity with aging (Bernet et al., 2014; Cosgrove et al., 2014; Sousa‐Victor et al., 2014). Cell‐intrinsic deficits that impact myogenic competence with aging are postulated to arise from a variety of factors, including depletion of the aged MuSCs due to increased asymmetric divisions within the heterogenous population (Bigot et al., 2015; Kuang et al., 2007), and alterations in cell‐cycle/differentiation signaling cascades, such as the p38/MAPK axis that negatively regulates MuSC self‐renewal through inhibition of *Pax7* and activation of *Myod1* (Palacios et al., 2010; Troy et al., 2012). However, the great majority of this work has been conducted in rodent models where proliferative persistence despite aging has been associated with long telomeres (Decary et al., 1997; Renault et al., 2002; Sacco et al., 2010; Zhu et al., 2007).

Certainly, among mammalian tissues, skeletal muscle is highly sensitive to the aging process and represents an impressive model in which to dissect the intrinsic and extrinsic components of age‐associated changes in tissue homeostasis and function (Etienne et al., 2020). A variety of grafting models have been developed to permit distinction of local from systemic influences on myogenic response. These have the merit over *ex vivo* culture models of retaining the *in vivo* context that is appropriate to the full biological phenomenon of muscle regeneration. Grafts of large pieces of structurally intact muscle have been shown to degenerate immediately but to regenerate on re‐establishment of vascular supply, using the remnants of the pre‐existing basement membranes as a guide to formation of a completely regenerated muscle of near‐normal structure. This paradigm was used to demonstrate that age‐related diminution of muscle regeneration in the rat is influenced predominantly by the age of the recipient rather than the donor (Carlson & Faulkner, 1989). More recently, with the availability of fully immunoincompetent mice as recipients, we have shown that skeletal muscle from facioscapulohumeral muscular dystrophy (FSHD) patients and unaffected relatives can regenerate effectively within the anterior tibial compartment of immunodeficient host mice and reproduce much of the gene expression profile of the donor genotypes (Zhang et al., 2014). These human donor grafts regenerate into muscle fibers containing close to 100% human myonuclei, together with a microvascular bed of dual mouse/human origin and innervation from the host anterior tibial nerves (Zhang et al., 2014). Here, we report further development of the xenograft model to successfully transplant muscle harvested postmortem from human cadavers that resulted in regenerates that are indistinguishable from that of grafts of living donors (Zhang et al., 2014). This is in accord with a report showing the rescue of viable and vigorously myogenic MuSCs from muscle several days postmortem (Latil et al., 2012). Although it has been suggested that myogenic cells remain viable within a muscle for around 2 weeks postmortem (Latil et al., 2012), the extent to which this population maintains full regenerative potential across an increasing postmortem interval (PMI) after death has yet to be investigated, where survival of other cell types, such as fibro‐adipogenic cells are increasingly understood as playing important accessory roles (Joe et al., 2010; Judson et al., 2013; Paylor et al., 2011; Uezumi et al., 2010; Yi & Rossi, 2011).

Our main objective was to provide an *in vivo* evaluation of the myogenic reserves of human skeletal muscle collected across a range of ages and PMIs. We hypothesize, here, that human muscle derived from either young, middle‐age, or old human cadavers would graft and regenerate equally effectively following the same time‐courses of proliferation, differentiation, and myofiber regeneration. Additionally, we hypothesized that MuSCs would maintain their myogenic potential after death at increasing PMIs. Our investigation revealed no measurable difference in human muscle regeneration across the range of ages investigated up to 78 years of age, and further, demostrated that MuSCs maintained their myogenic capacity even within muscles grafted up to 11 days postmortem. We conclude that the loss of muscle mass with increasing age is not attributable to any intrinsic loss of myogenicity of the MuSCs and is most likely a result of persistent detrimental alterations in the multi‐cellular muscle and systemic environments. Our results contest the notion that intrinsic loss of proliferative or differentiative functions by the MuSC population in aged muscle is the major factor behind loss of muscle mass in aged and cachexic individuals.

## 2 RESULTS

### 2.1 Xenograft model of human muscle satellite cell‐mediated regeneration

In this study, our primary objectives were to comprehensively evaluate the influence of human age and PMI on the regenerative capacity of human MuSCs using the model of a human xenograft into an immunodeficient mouse. Human postmortem muscle samples were procured with approval by the Children's National Hospital Institutional Review Board in collaboration with regional medical centers. Samples were described only by age, sex, race, and date/time of death and autopsy. Exclusion criteria for human samples included only patients with blood‐borne infectious diseases (i.e., HIV, Hepatitis) or neuromuscular diseases. Xenograft transplantation was performed in accordance with procedures in our previous development of this model (Zhang et al., 2014). We obtained a total of 13 human muscle samples from both males and females ranging from 28 to 91 years of age (Table 1). Human muscle samples were isolated predominantly from the psoas muscle, but also included samples of abdominal and rectus femoris muscles (Table 1). In brief, human donor muscle samples were dissected at autopsy under sterile conditions and transported to Children's National Hospital. They were trimmed into multiple ~8 mm length strips, approximating the size of the mouse tibialis anterior muscle, and transplanted into the anterior compartment of NOD. Cg‐*Rag1^tm1Mom^ Il2rg^tm1Wjl^*/SzJ (NRG) immunodeficient mice (Pearson et al., 2008; Zhang et al., 2014). Each single human muscle strip was sutured to the distal and proximal tendons of the mouse extensor digitorum longus muscle and the graft site was closed with surgical glue and wound clips.

**TABLE 1:** Human muscle samples and xenograft study results based on donor age and postmortem interval

Age	Sex	Race	Muscle	Postmortem Interval (PMI)	Success rate (*n* per PMI)
28 years	Female	African American	Psoas	4 days, 8 days, 12 days	0/8 (4 days), n/a (8 days), n/a (12 days)
36 years	Male	Caucasian	Psoas	5 days, 7 days	2/8 (5 days), 1/9 (7 days)
41 years	Male	African American	Psoas	3 days, 5 days	0/5 (3 days), 0/1 (5 days)
54 years	Male	Caucasian	Psoas	2 days, 4 days	1/2 (2 days), 1/3 (4 days)
58 years	Male	African American	Psoas	3 days, 6 days	2/2 (3 days), 4/4 (6 days)
66 years	Female	Not specified	Abdominal	4 days, 11 days	8/10 (4 days), 7/11 (11 days)
68 years	Male	Not specified	Psoas	5 days, 7 days	0/5 (5 days), 0/4 (7 days)
70 years	Female	Caucasian	Psoas	3 days, 5 days	0/2 (3 days), 0/1 (5 days)
70 years	Female	Not specified	Psoas	5 days, 6 days, 7 days	0/1 (5 days), 0/3 (6 days), 0/2 (7 days)
75 years	Male	Not specified	Abdominal	2 days, 3 days, 6 days	4/4 (2 days), 2/2 (3 days), 3/3 (6 days)
77 years	Male	Not specified	Abdominal	2 days, 6 days	2/3 (2 days), 0/2 (6 days)
78 years	Male	African American	Rectus Femoris	11 days	4/6 (11 days)
91 years	Male	Not specified	Psoas	2 days, 9 days, 16 days	0/5 (2 days), 0/5 (9 days), n/a (16 days)

Note

Description of the age, sex, race, muscle sample, postmortem interval (PMI), and rate of success for all human cadaver samples investigated in the current study.

Graft sites were examined 3‐ or 6‐week post‐engraftment to investigate efficiency of muscle regeneration with donor age and the PMI between death and xenograft transplantation. Overall, successful regeneration of the human muscle was found with 7 of the obtained human muscle samples in this study, at PMIs between 2 and 11 days (Table 1). Successful regeneration of the human muscle samples was confirmed by immunostaining for human membrane protein spectrin and nuclear protein lamin A/C with human‐specific monoclonal antibodies (Figure S1a). While not all muscle grafts successfully regenerated to form myofibers within the host, in many of these samples we did find evidence of human spectrin and lamin A/C that localized within areas of connective tissue adjacent to regenerated host muscle (Figure S1b). In some cases, failure to regenerate was associated with infection attributable to contamination during isolation of the donor muscle or during the engraftment procedures. Assessment of regenerative success (i.e., number of human myofibers and area) was complicated by variation along the length of the regenerate (Figure S1c).

### 2.2 Grafted human muscle satellite cells maintain myogenic capacity irrespective of age

**FIGURE 1:**
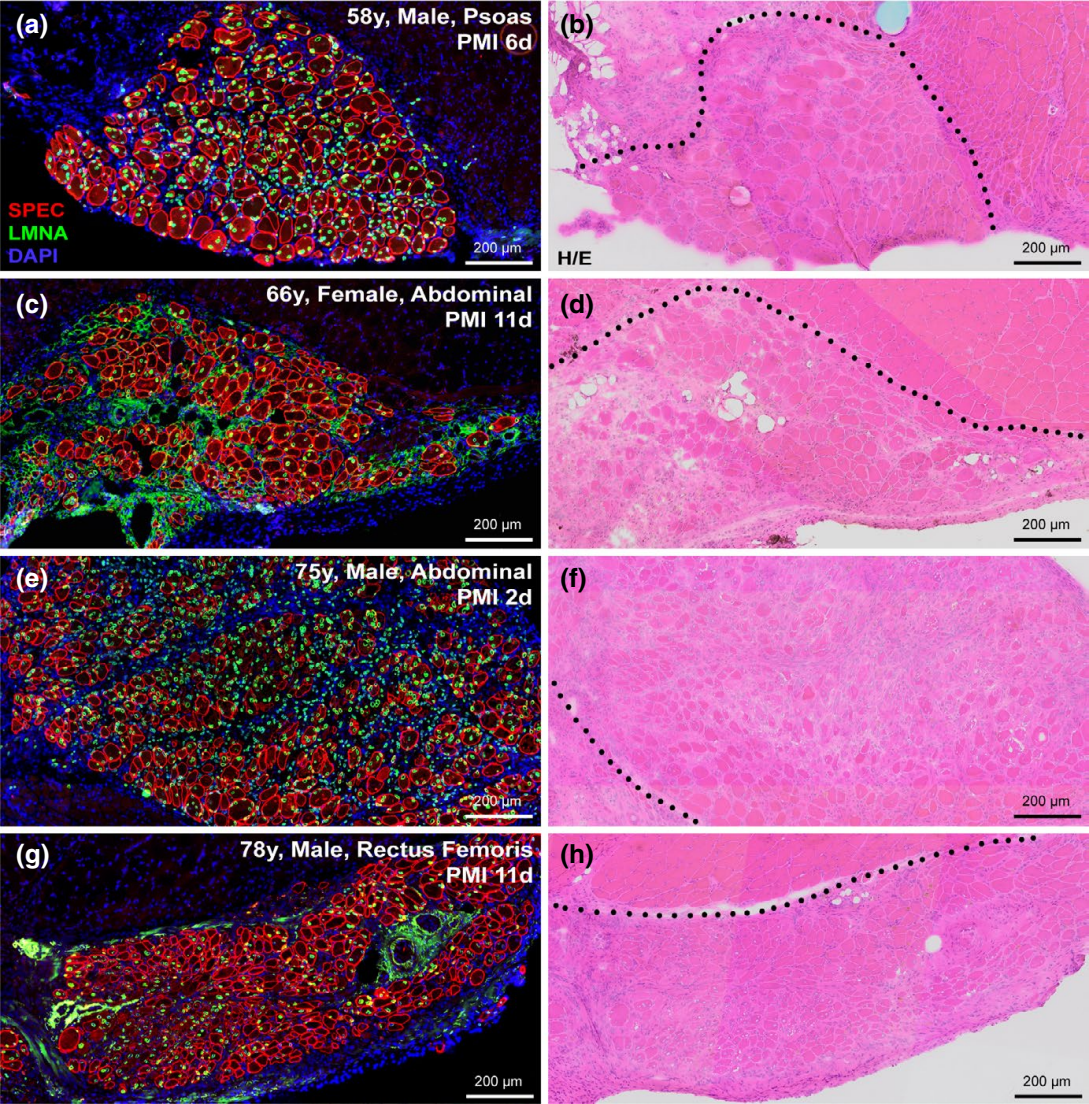
Myogenic capacity of human MuSCs is uncompromised despite aging. (a‐h) Human skeletal muscle regeneration following postmortem muscle tissue engraftment from human cadavers of various ages and PMIs. Immunostaining for human‐specific spectrin (SPEC, red), and lamin A/C (LMNA, green) proteins identify regions regenerated from dormant human MuSCs within the grafted tissue, while DAPI (blue) mark the entire muscle tissue section for reference (a, c, e, g). Human muscle regeneration shown here from transplanted human cadaver muscle at the indicated PMI; 58‐year‐old (a, b; male, psoas), 66‐year‐old (c, d; female, abdominal), 75‐year‐old (e, f; male, abdominal), and 78‐year‐old (g, h; male, rectus femoris). Histology of adjacent serial sections performed by H/E staining shows regrowth of both human and mouse muscle tissue (delineated by black dotted line) harvested from anterior tibial compartment 3‐week post‐engraftment (b, d, f, h). Scale bars represent 200 μm (a‐h).

To first investigate the influence of age‐related sarcopenia on the regenerative capacity of human MuSCs, we performed xenograft transplantation of postmortem human muscle samples from individuals ranging from 28 to 91 years of age. Analysis of xenografts at 3‐ or 6 weeks following engraftment indicated successful regeneration across the range of donor ages, with plentiful regenerated myofibers of human origin being identified at sites of grafts from donors of 36, 54, 58, 66, 75, and 78 years of age (Table 1). Further, the most robustly regenerative human muscle samples consistenty yielded both the the greatest numbers of regenerated human myofibers and the largest contiguous areas of regenerated fibers (Figure 1a‐h; Figure S2) at 2 or more graft sites. These results were found across the range of ages (58‐, 66‐, 75‐ and 78‐year‐old) of human samples. Thus, our xenograft model revealed no compromised myogenic capacity of human MuSCs with age (Figure 1a‐h; Figure S2). Across the range of ages tested, we also noted no differences in initial rate of regeneration or subsequent maturation of these human myofibers over the first 3 weeks post‐engraftment (Figure 1a‐h). Despite the lack of evidence of regenerated human myofibers in our 28‐ and 91‐year‐old cohorts that correspond to minimum/maximum ages within our associated study (Table 1), our data does not suggest any deficit in regenerative capacity of human MuSCs associated with increasing human donor age.

**FIGURE 2:**
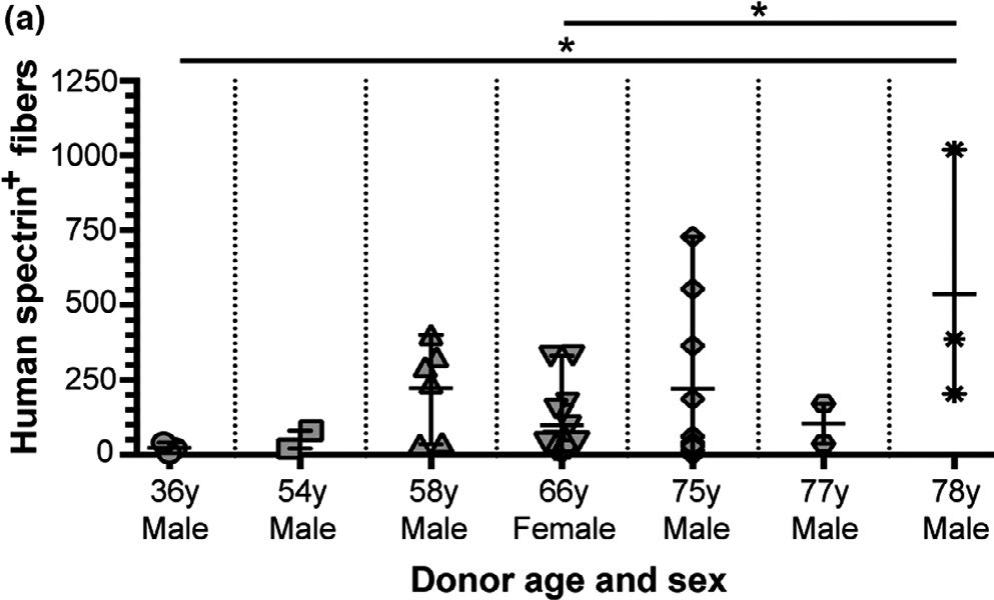
Assessment of human muscle regeneration by donor age and sex. (a) Regenerated human skeletal muscle fibers were quantified based on proper localization of human‐specific spectrin and lamin A/C proteins. Data represented as scatter‐plot with mean and standard deviation (SD) for all successfully regenerated cadaver samples as indicated in Table 1. Statistical analyses performed by one‐way ANOVA with multiple comparisons; *p < 0.05.

Analysis of engraftment efficacy by sex or race also did not reveal any major differences in the survival or regenerative capacity of human MuSCs. Of the 13 human muscle samples collected, 4 were from female cadavers while 9 were from male cadavers. While we failed to find evidence of regeneration in 3 of 4 female samples, the 66‐year‐old female sample was closely comparable to the successful male grafts in terms of its regenerative capacity (Table 1; Figure 2). Taken together with the relatively short PMI tested for all female samples (PMIs ranging 2–5 days), it would suggest potential issues with the health status of the muscle sample (contamination or compounding illness) or surgical procedure rather than any link to sex. Of the 9 male muscle samples evaluated in our study, we failed to find evidence of regeneration in 3 of the xenograft surgeries performed; however, we found that all three 70 to 80‐year‐old male samples regenerated well in our xenograft model (Table 1; Figure 2). Stratification of the data by race also did not point toward any apparent link with regenerative capacity. In our study, we evaluated 4 African American muscle samples (2 of which regenerated) and 3 Caucasian muscle samples (2 of which regenerated), while 6 were not specified in terms of donor race (Table 1; Figure 2). The number of samples received limits our evaluation in terms of this demographic; however, we see no indication of any major differences based on sex or race in this study.

### 2.3 Myogenic capacity is preserved with increasing postmortem intervals

Skeletal muscle biopsies from postmortem human cadavers have been shown to exhibit severe loss of normal myofiber structure over a range of PMIs (Latil et al., 2012). In contrast, MuSCs can adopt a quiescent state and retain their regenerative capacity postmortem (Latil et al., 2012). This intriguing cellular adaptation within the seemingly hostile, necrotic environment of a postmortem cadaver muscle occurs under conditions of anoxia and nutrient deprivation (Latil et al., 2012). Furthermore, the postmortem isolation of MuSCs at intervals from both murine and human muscle demonstrated retention by quiescent satellite cells of proliferative and regenerative capacities (Latil et al., 2012). Despite the compromised structure of postmortem muscle tissue, the myogenic cells have been shown to have a selective survival advantage compared to other cell types postmortem (Latil et al., 2012).

Another of our major primary objectives was to investigate retention of regenerative capacity of human MuSCs maintained under anoxic conditions for extended periods. Cadaver muscle samples were received at 2–11 days postmortem and stored in Dulbecco's Modified Eagle's Medium (DMEM) at 4℃ until grafting—the total time being defined as our PMI. Histological examination of our postmortem muscle samples made at the time of engraftment, revealed substantial loss of both myofiber structure and nuclear karyolysis and karyorrhexis across the range of PMIs of 4–16 days (Figure S3). For PMIs beyond 10 days, there is a clear loss of myofibers throughout the sample, while their myonuclei and the various interstitial nuclei become far less conspicuous (Figure S3a, f, l, m).

**FIGURE 3:**
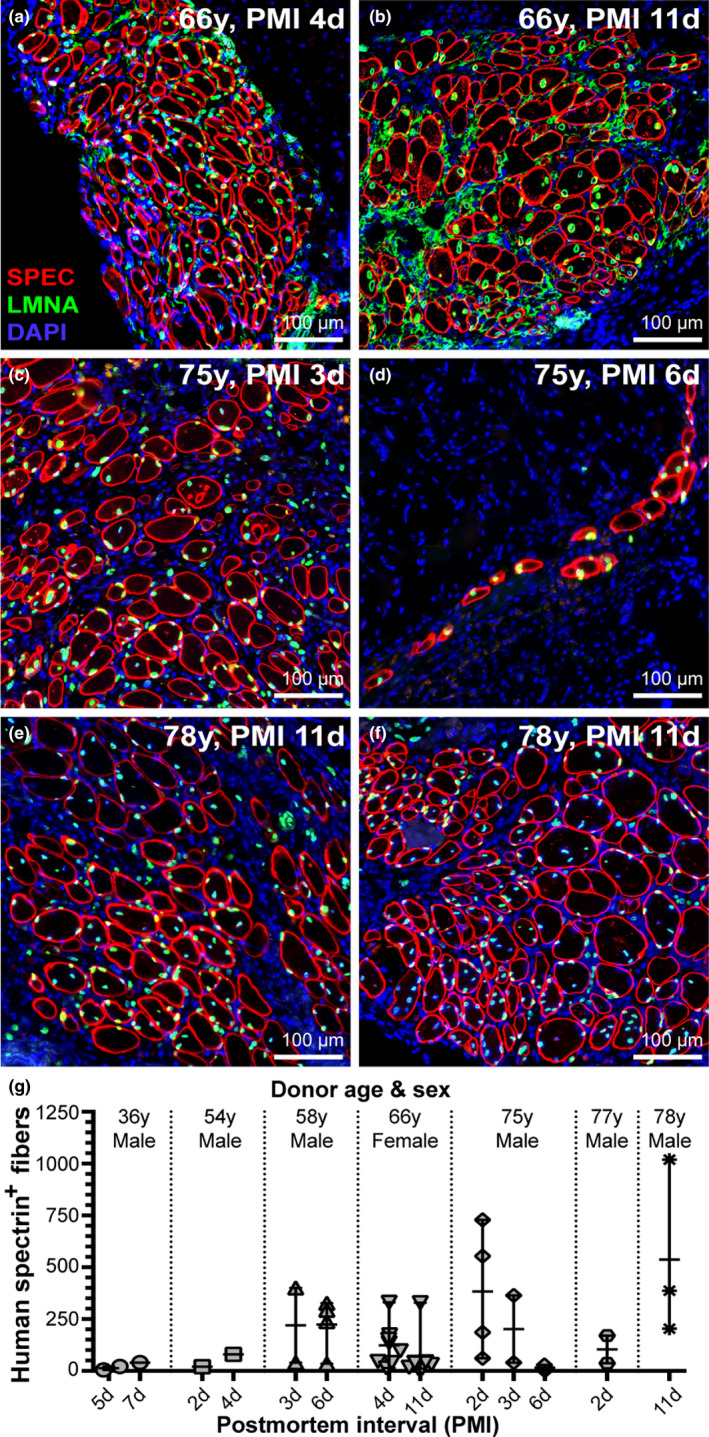
Resiliency of grafted human MuSCs after prolonged postmortem dormancy. (a‐g) Assessment of human muscle regeneration at escalating PMIs. Comparisons at two PMIs for the 66‐year‐old sample (a, b; PMI 4 days and 11 days) and 75‐year‐old sample (c, d; PMI 3 days and 6 days) are shown. Successful regeneration after 11 days postmortem is demonstrated in two xenografts for the 78‐year‐old sample indicating resiliency of human MuSCs despite prolonged PMI when maintained under anoxic conditions (e, f). Representative images indicate no apparent decline in MuSC survival or regenerative capacity out to 11 days postmortem (b, e, f, g). (g) Regenerated human skeletal muscle fibers were quantified and represented as scatter‐plot with mean and SD for all successfully regenerated cadaver samples stratified by PMI as indicated in Table 1. Statistical analyses were performed (when permitted by n‐values) by non‐parametric, Mann–Whitney test to assess differences between PMIs for a given sample.

In the 5 of our 7 human cadaver muscle samples that successfully regenerated *in vivo*, we grafted at a number of PMIs whenever possible, depending on the size of the sample and the availability of suitable recipient mice. In all human muscle samples that successfully regenerated *in vivo*, we found no apparent decline in the regenerative capacity of human MuSCs with extended PMIs (Figure 3a‐g). Most notably, longitudinal assessments of the 66‐year‐old human muscle samples at PMIs of 4 and 11 days indicated preserved myogenic integrity with regard to the amount of muscle regeneration we observed in this model (Figure 3a,b,g). In the case of the 75‐year‐old sample, we found low numbers of regenerated human fibers in our third engraftment attempt at a PMI of 6 days but the low n‐value leaves us unable to evaluate the extent of sampling error (Figure 3c,d,g). Further, assessment of the 78‐year‐old sample at a single PMI of 11 days yielded our most conspicuous regenerates of human muscle, indicating no significant loss in regenerative capacity with either advanced age or prolonged PMI (Figure 3e,f,g). In both these cases, PMIs out to 11 days were found to regenerate efficiently *in vivo* (Figure 3g). Overall, our data stratified by PMI for all successful human samples indicate no conspicuous loss of MuSC regenerative capacity out to nearly 2‐week postmortem, and suggests that postmortem muscle tissue and its resident MuSCs could provide a valuable tool for future experimental and translational regenerative studies of human muscle.

### 2.4 Regenerated human muscle maintained growth and fiber size across all ages

**FIGURE 4:**
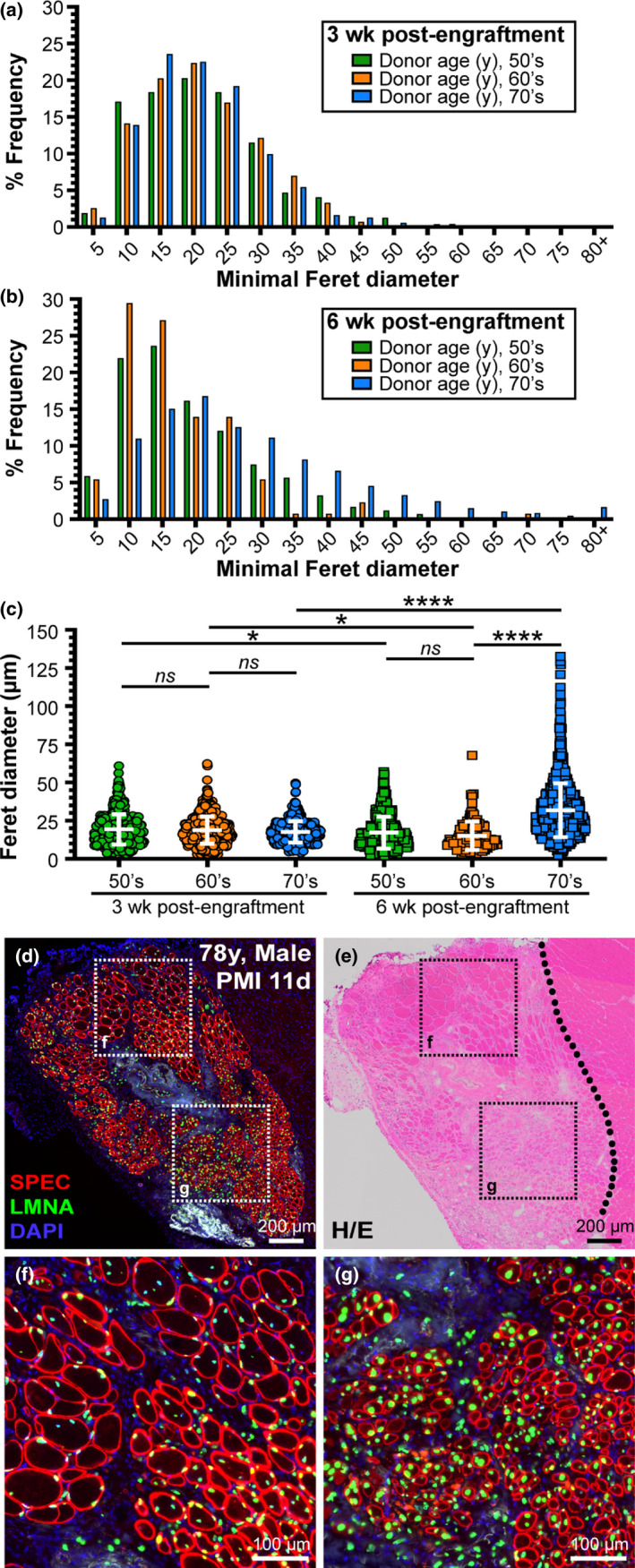
Analysis of muscle fiber growth post‐engraftment. Myofiber diameter data were stratified by age in decades (i.e., 50‐, 60‐, and 70‐year‐old samples). (a) Minimal Feret diameter quantified from samples stratified by age bracket harvested 3 weeks post‐engraftment (50's, *n* = 2 samples and 468 fibers; 60's, *n* = 1 sample and 815 fibers; 70's, *n* = 3 samples and 1884 fibers). (b) Minimal Feret diameter quantified from samples stratified by age bracket harvested 6 weeks post‐engraftment (50's, *n* = 2 samples and 829 fibers; 60's, *n* = 1 sample and 130 fibers; 70's, *n* = 3 samples and 2167 fibers). (c) Scatter‐plot showing mean with SD at both 3‐week and 6‐week post‐engraftment for all cohorts. (d, e) Human skeletal muscle regeneration following postmortem (PMI, 11 days) muscle tissue engraftment from 78‐year‐old human cadaver 6‐week post‐engraftment. Immunostaining for human‐specific spectrin (SPEC, red) and lamin A/C (LMNA, green) proteins (d) indicate the extent of regeneration in this “high‐responder” xenograft, while H/E staining (e) shows the boundary of human muscle regeneration adjacent to the regeneration mouse muscle tissue (delineated by black dotted line). (f, g) Inlays represent areas of mature fibers marked predominantly with peripheral nuclei labeled with human lamin A/C (f) or newly regenerated, centrally nucleated fibers as indicated by the position of lamin A/C‐labeled myonuclei (g). Scale bars represent 200 μm (d, e) and 100 μm (f, g). Statistical analyses performed by one‐way ANOVA with multiple comparisons; ns, not significant, *p < 0.05; ****p < 0.0001.

Although we found no evidence of myogenic deficit with aging, we looked for deficits in myofiber growth between the 3‐ and 6‐week post‐engraftment cohorts. To assess this, we measured the minimal Feret diameter (MFD) of all human fibers after 3‐ and 6‐week post‐engraftment and stratified the data by age in decades (i.e., 50‐, 60‐, and 70‐year‐old samples; Figure 4a‐c). By using human‐specific spectrin membrane immunostaining, we were able to differentiate muscle fibers derived from human MuSCs versus those derived from mouse MuSCs. While the size distribution of fibers from each cohort is strikingly similar at 3 weeks post‐engraftment, the distribution after 6 weeks demonstrates an increase in small‐diameter fibers that may be attributable to a second wave of regeneration, in addition to an increase in size of the more mature myofibers when compared to the data collected at 3‐week post‐engraftment (Figure 4a‐g). The median MFD was very similar at 3 weeks post‐engraftment in all groups (~20 µm), and overall 60% of the total fibers were less than 20 µm in diameter while 40% were greater than 20 µm. In contrast, at 6 weeks post‐engraftment, while the combined median MFD of all groups was also ~20 µm (53% of the total fibers were less than 20 µm in diameter while 47% were greater than 20 µm), the median for each group ranged from ~13 to 21 µm. The MFD from both the 50‐ and 60‐year‐old cohorts was just slightly smaller on average at 6 weeks vs. 3 weeks post‐engraftment due to increased presence of clustered small‐diameter, newly regenerated human fibers less than 15 µm across (Figure 4c), again suggestive of persistent regeneration. In contrast, the MFD of the 70‐year‐old cohort was on average 27.6% greater at 6 weeks than at 3 weeks post‐engraftment due to the presence of clusters of larger size fibers ranging from 50 to 130 µm in diameter in two of our 78‐year‐old xenograft samples (Figure 4d‐g, Figure S4). This finding is epitomized by one of our more successful xenografts (78‐year‐old human sample), that produced 537 human myofibers across multiple successful transplants, and a study‐wide maximum of 1019 regenerated human myofibers (Figure 4d‐g, Figure S4). Observations for these xenografts at 6‐weeks post‐engraftment revealed numerous clusters of large, mature myofibers with a predominance of human myonuclei located in a juxta‐sarcolemmal position (Figure 4d,f; Figure S4). The distinctive difference in size and myonuclear positioning in these fibers, together with their segregation from the groups of smaller fibers, is most simply explained as the result of successful innervation of these fibers that further promotes their growth. This explanation is consistent with our findings of motor endplates in our earlier study (Zhang et al., 2014). Additionally, in other areas within these same xenografts, we located numerous, small‐diameter myofibers with centrally placed myonuclei suggesting a more recent bout of regeneration perhaps involving fiber branching (Figure 4d,g). The patches of small centrally nucleated fibers seen at 6 weeks were localized to zones around the blocks of mature fibers and were intermixed with inflammatory or interstitial connective tissue associated with surgery. Together, these results imply a persistence of regeneration and growth for at least 6 weeks beyond the initial period of regrowth that occurs during the first 3 weeks following engraftment.

### 2.5 Sustained expression of myogenic transcription and regulatory factors indicate regeneration after initial regrowth period

**FIGURE 5:**
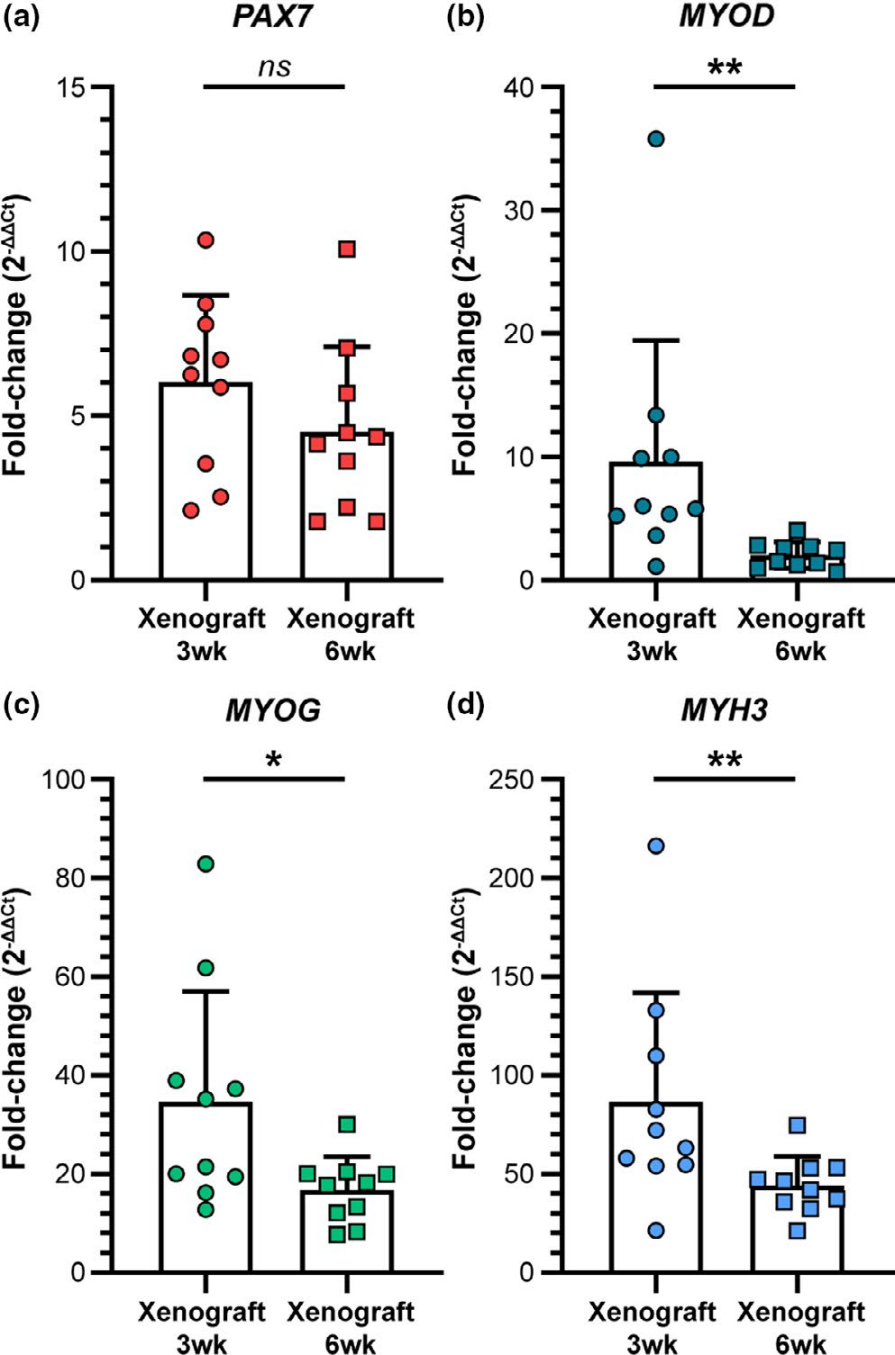
Gene expression analyses of human myogenic markers over time following engraftment. (a‐d) Relative gene expression reported as normalized fold‐change for human‐specific myogenic mRNA transcripts *PAX7* (a), *MYOD* (b), *MYOG* (c), and *MYH3* (d) for xenograft samples collected 3‐ and 6‐week post‐engraftment. Raw qRT‐PCR data were normalized to human‐specific house keeping gene, HRPT, and non‐regenerated human negative control muscle to calculate ΔCt and ΔΔCt, respectively. Normalized gene expression fold‐change was calculated using the formula, 2^−(ΔΔ^ *^C^* ^t)^. Statistical analyses performed by two‐tailed, non‐parametric, Mann–Whitney test; ns, not significant; *p < 0.05; **p < 0.01.

To test whether persisting bouts of muscle regeneration contributed to the significant number of spatially clustered, small‐diameter fibers observed 6 weeks post‐engraftment (Figure 4), we investigated longitudinal gene expression levels by qRT‐PCR for myogenic transcription factors, *PAX7* (Figure 5a), *MYOD* (Figure 5b), and *MYOG* (Figure 5c), and embryonic myosin heavy chain, *MYH3* (Figure 5d), normalizing our Ct values to those xenografts that failed to regenerate human muscle fibers. We observed up‐regulation of human‐specific *PAX7*, *MYOD*, *MYOG*, and *MYH3* transcripts in successfully regenerated xenografts at both 3‐ and 6‐week post‐engraftment compared to human, non‐regenerating controls. Unsurprisingly, we found a reduction in myogenic transcripts between 3 weeks and 6 weeks post‐engraftment, yielding significantly lower expression of *MYOD*, *MYOG*, and *MYH3* at the later time point. Nonetheless, even the “low” transcript levels found 6 weeks post‐engraftment, were still present in abundance and heavily overlapped the overall range of those detected at 3 weeks post‐engraftment, indicating a persistence of active myogenesis at both 3 and 6 weeks post‐engraftment (Figure 5a‐d). The seemingly low expression of *MYOD* at 6 weeks is attributable to its relatively transient expression during myogenesis, relative to the duration of *MYOG* and *MYH3* expression in terminal myoblasts and the early developing fiber, respectively (Figure 5b‐d). Persistence of *MYOG* and *MYH3* transcripts over both time points evaluated (Figure 5c‐d) may indicate additional rounds of myogenesis following the early regrowth phase post‐engraftment, aligning well with our reports of numerous small‐diameter, centrally nucleated fibers at both time points (Figure 4).

Overall, these data support our conclusion that human muscle regeneration persists for at least 6 weeks post‐engraftment and that the human muscle remains relatively myogenic beyond the initial regrowth phase at both time points evaluated in this study.

### 2.6 Human myogenic cells migrate and regenerate beyond the initial graft site

**FIGURE 6:**
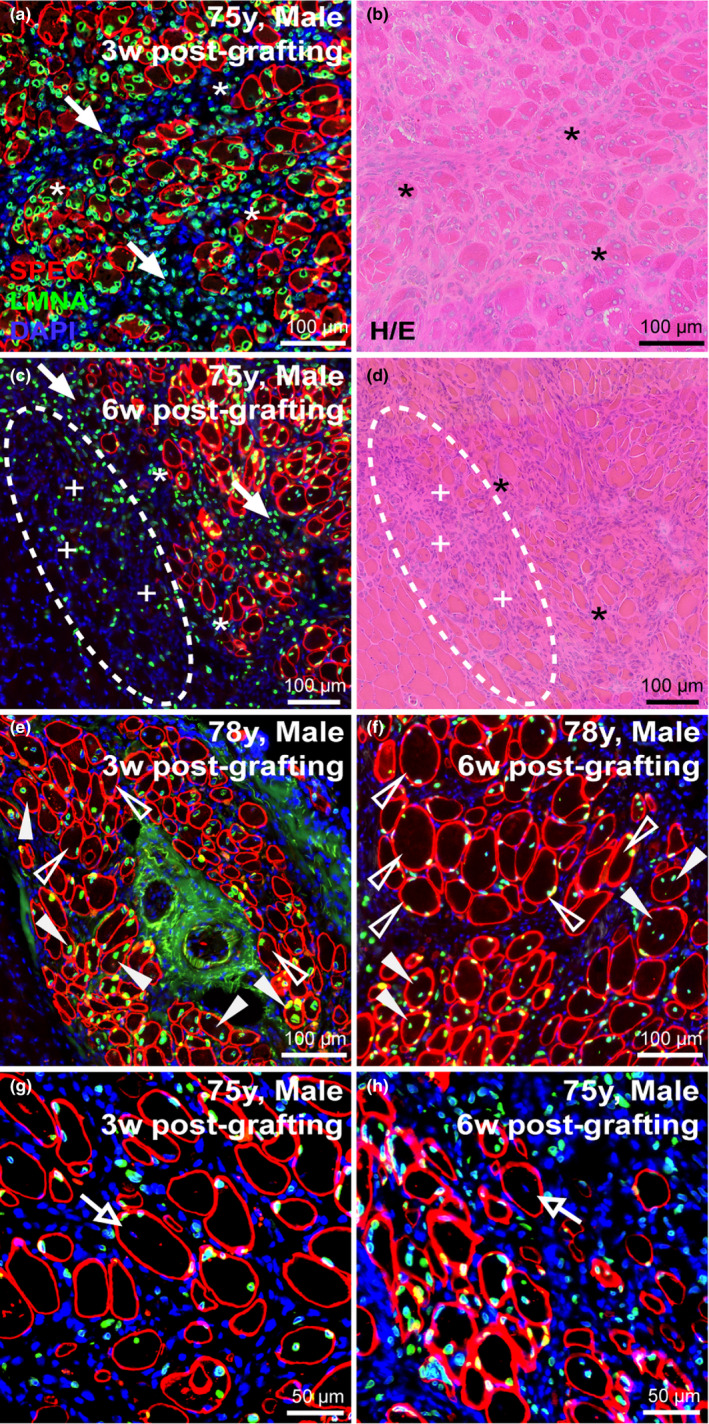
Myonuclear localization and migration post‐engraftment. (a‐d) Longitudinal assessment of 75‐year‐old sample harvested either 3‐week (a, b) or 6‐week (c, d) post‐engraftment assessed by immunostaining human‐specific protein (a, c) and H/E histology (c, d). Immunostaining for human‐specific spectrin and lamin A/C indicate frequency of central nucleation or peripheral nucleation in regenerated human myofibers 3‐week (a) or 6‐week (c) post‐engraftment. Prevalence of interstitial mononuclear cell types indicated at intervals post‐engraftment (solid arrows) and their increased presence beyond the boundary of mature human myofibers prevalent in samples 6‐week post‐engraftment (c, d; dotted circular region). Within these new regions low‐intensity spectrin membrane staining was observed in small‐diameter fibers (c, d; indicated by “+” signs adjacent to fibers). Asterisks (*) denote muscle orientation between serial cross‐sections (a‐d). (e, f) Longitudinal assessment of regeneration and nuclear localization after regeneration of the 78‐year‐old sample harvested either 3‐week (e) or 6‐week (f) post‐engraftment. Solid arrowheads indicate centrally nucleated fibers, while empty arrowheads indicate myonuclei that have adopted a peripheral position within the myofiber (e, f). (g, h) Myonuclei of mouse origin (negative for human lamin A/C) observed within regenerated human spectrin‐positive fibers; open arrows identify murine central myonuclei. Scale bars represent 100 μm (a‐f) or 50 μm (g, h).

Intriguingly, microscopic analysis of the regenerated human muscle at both 3‐ and 6‐week post‐engraftment indicate the persistence of human mononuclear cells (lamin A/C‐positive) within the interstitial space between the regenerated human myofibers (Figure 6a‐d; *solid arrows*). These human cells were localized strictly between zones of human and adjacent mouse muscle (Figure 6a‐d). This may indicate the survival and expansion of various cell types within the regenerated human muscle that promote MuSC‐mediated regeneration (Blau et al., 2015; Lukjanenko et al., 2019). Additionally, with prolonged fiber growth after engraftment (i.e., 6 weeks after transplantation), we noted a reduction in the interstitial space between human fibers and the diminution in number of these interstitial mononuclear cells (Figure 6a‐f), as the inflammatory and fibrotic processes resolved over time. Further, in grafts examined 6‐weeks post‐engraftment, we observed populations of human interstitial cells beyond the borders of human myofibers identified by human lamin A/C (*defined by dashed circular region*), as well as some low‐intensity spectrin staining in what appear to be small‐diameter fibers forming within this same region among the human lamin A/C‐labeled mononuclear cells (Figure 6c,d; *indicated by ‘+’ signs*). These data together with our findings of both small‐ and large‐diameter myofiber clusters at 6 weeks post‐engraftment (Figure 3d‐g) indicate continuing expansion of MuSCs and myofiber regeneration, even from our oldest human samples, for at least 6 weeks after transplantation.

### 2.7 Human myonuclei adopt positions subjacent to the sarcolemma following regeneration

In the mouse, central nucleation provides a much used identifier of regenerated myofibers in skeletal muscle. Interestingly, in line with our previous observations (Zhang et al., 2014) the myonuclei of regenerated human myofibers had largely adopted a peripheral position subjacent to the sarcolemma (Figure 6e,f; *open arrowheads)* by 3 weeks and increasingly so in samples harvested 6‐weeks post‐engraftment. We previously estimated that some 80% of regenerated mouse muscle fibers contain centrally positioned nuclei in cross sections (Novak et al., 2017). By contrast, in the newly regenerated, lamin‐positive human fibers, central nucleation represented only a minor fraction of myonuclei marked by human lamin A/C at 6‐week post‐engraftment (Figure 6f; *solid arrowheads*). This would either indicate differences in myonuclear domain and spatial distancing in human fibers compared to that in mice or, perhaps, changes in the rates of migration between the central and peripheral positions following myofiber repair or complete regeneration.

### 2.8 Satellite cells of mouse origin fuse into regenerating human myofibers

One further point of interest arising from microscopic inspection of the border between regenerated human and mouse muscle tissue were rare observations of human myofibers containing central nuclei unmarked by human lamin A/C. While the vast majority of regenerated human myofibers immunostained for both human spectrin and lamin A/C, these “mosaic” myofibers likely resulted from the fusion of mouse with human cells during myogenesis (Figure 6g,h). This was seen only in samples collected 6‐week post‐engraftment and suggests that mouse MuSCs migrated and fused with actively regenerating human myofibers yielding “mosaics” comprised of both human and mouse myonuclei.

## 3 DISCUSSION

Debilitating effects of waning tissue function with age are currently a central medical issue in the context of aging populations in most “developed” countries, with loss of mass and strength of skeletal muscle in the vanguard. Unsurprisingly therefore, identification of the biological mechanisms behind maintenance of muscle tissue has assumed increasing prominence as a topic of debate and research. Medawar's seminal essays exploring the fundamental nature of aging (Medawar, 1946, 1952), point to a model that predicts an accumulation of functional defects that manifest too late in the life of an individual to significantly affect fecundity and thus fail to be eliminated, by natural selection, from the gene pool. It is to be expected therefore that aging would be a multifactorial consequence of a spectrum of genes operating adversely toward the end of the individual's lifespan, with few specifically definable culprits. For skeletal muscle, a prominent issue is its loss of mass beyond the 4th decade, centering on the idea of failure of the mechanisms of muscle maintenance, commonly postulated to involve myogenic replacement of muscle lost during day‐to‐day damage. One level of discussion around this point centers on the extent of such a maintenance function during day‐to‐day activity. The main evidence on this point, the progressive reduction in telomere length and of *in vitro* proliferative potential in MuSC, argues strongly against any major implicaton of MuSC activity beyond adolescence except in myopathic individuals (Decary et al., 1997; Renault et al., 2000).

**FIGURE 7:**
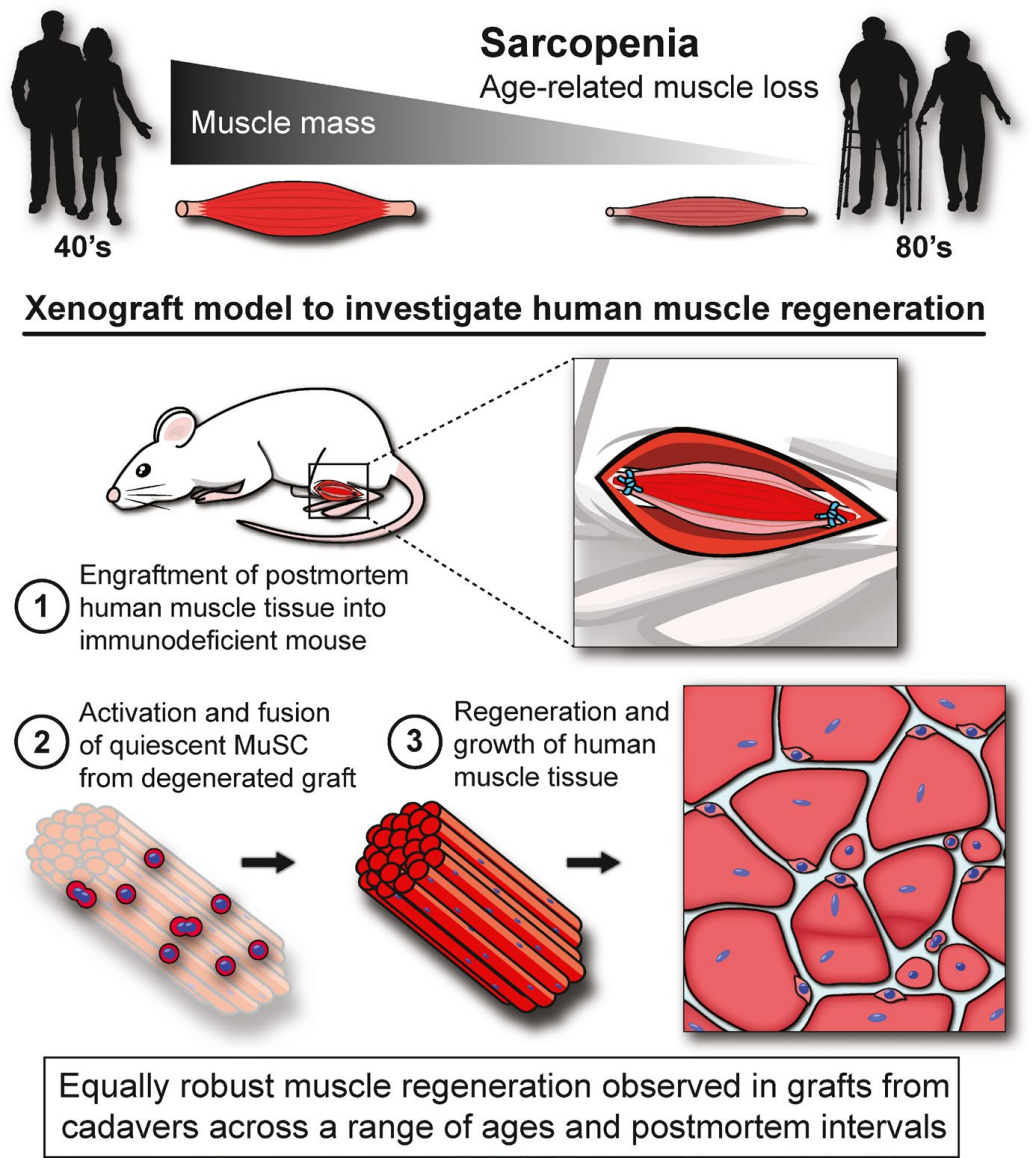
Xenograft model to investigate human muscle regeneration. Schematic demonstrating the xenograft model used to investigate the impact of aging on myogenic capacity of human muscle during regeneration *in vivo*. Human cadaver muscles were surgically grafted into the anterior tibial muscle compartment of immunodeficient mice and allowed 3–6 weeks for regeneration (1). During this period, quiescent human MuSC within the degenerated postmortem tissue (2) underwent activation and fusion to form completely regenerated human muscle tissue of normal structure despite advanced donor age and prolonged PMIs (3).

Nonetheless, the notion of a functional deficit in the MuSC population remains a leading candidate to explain age‐related sarcopenia, a debate centering on whether any demonstrable dysfunction of this cell in the aging muscle is attributable to some intrinsic property of the aging MuSC itself or on its functional competence in an environment degraded by the ravages of time and subliminal pathology. In the aging mouse, the main experimental model, there is strong evidence for age‐related distrubances of the mechanisms that mediate the activation and of their return to dormancy that, between them, would tend to hamper its role in regeneration. But we lack models in which to establish a direct link of such molecular biological data to a measurable defect in regeneration *in vivo*. Functional defects detected in vitro in extracted myogenic cells are conducted in the absence of the plethora of complexities of the *in vivo* situation, while translation of defects in regeneration of mouse muscle are open to doubts as to the relevance to humans of the fundamentally different muscle cell biology and inflammatory milieu in the mouse. Further, while the *in vitro* system allows for the assessment of a plethora of outcomes on isolated cells, understanding the function of MuSC *in vivo*, with its other niche components, allows for a more biologically revelant evaluation of their myogenic potential within the tissue itself. Therefore, here we used our established xenograft model as a method to evaluate the *in vivo* function of aged MuSCs into a youthful environment (Figure 7). Additionally, work from others evaluated different metrics of myogenicity of isolated MuSCs *in vitro* to demonstrate their viability and capacity over different PMIs (Latil et al., 2012).

In humans, the evidence for an intrinsic defect in aged MuSC consists of counts of the numbers of MuSC present in muscle samples and of their activity in vitro. Definitive data relating MuSCs to age in man, derives from a full stereological analysis and describes a halving of MuSC number per volume (cm^3^) in human vastus muscle between young and old individuals (Sajko et al., 2004). These data should be viewed in the perspective that the difference might, in principle, be accommodated by a single round of cell division, and further information on the behaviour of these cells is needed if we are to build a coherent picture of the effects of age on muscle regeneration. Here, we have attempted to set up the closest situation we could devise to an in vivo bout of human muscle regeneration as a means to evaluate the myogenic potential of aged human muscle. As is commonly the case with human research, technical and practical issues obtruded somewhat and we were unable to gather data across as great a range of ages as we had envisaged. Additionally, while we originally sought to evaluate the effects of different PMIs on the function of satellite cells, technical shortcomings limited our ability to fully evaluate this objective. It also proved difficult, with several sites of autopsy, to standardize the interval between death and performance of the graft, so we have included this experimental variable in the experimental design. Interestingly, our data show no obvious effect of PMI on the myogenic performance of the grafts. The implication that the muscle stem cell compartment within the MuSC population is highly resistant to the anoxic conditions of postmortem muscle accords with a previous demonstration of persistence of myogenic cells in such an environment (Latil et al., 2012). This finding resolves an old debate regarding whether the source of myogenic cells in the anoxic center of large muscle grafts is due to survival or by immigration from neighboring less anoxic regions (Phillips et al., 1987; Schultz et al., 1988). Clearly, the ability of MuSCs to survive anoxic conditions would enhance their value for recovery from large regions of traumatic muscle injury. We were also surprised to have had difficulty obtaining autopsy samples from younger adults, which would have provided a better comparison across the ages—perhaps this was due to the factors that lead to the decision to perform an autopsy (e.g., cause of death).

Nonetheless, our data show that the very oldest human muscles still contain a population of myogenic cells that were able to reconstruct a substantial volume of well‐formed muscles with only minimal interstitial fibrosis (Figure 7). This requires well‐coordinated choreography of the serial stages of myogenesis in a xenogeneic systemic environment where the inflammatory and vascular systems, as well as cytokine and hormonal signals, differ in detail from those that would operate *in vivo* in human, suggesting that much of the underlying regulation is local and intrinsic to the graft (Figure 7). The near‐normal architecture is probably attributable in large part to the persistence of the structural cues of basement membrane remnants. However, we cannot exclude effects of persistent non‐myogenic cells, such as fibroadipocytes of human origin, and our previous work has shown that some of the microvascular structure is derived from human cells present in the graft (Zhang et al., 2014). Whatever the case, our data show that even aged human muscles remain fully competent to undertake extensive myogenesis in a friendly environment (Figure 7). Closer inspection revealed that our regenerated grafts retained their myogenic function far beyond the initial phase of regrowth after surgery, where they maintained heightened expression of terminal markers of myogenesis (*MYOG*), and myofiber development (*MYH3*), outward for at least 6 weeks post‐engraftment. These data, in parallel with our histological examinations that revealed numerous clusters of small‐diamater, centrally nucleated fibers in grafts at this time point, are indicative of subsequent bouts of MuSC expansion and persisting myofiber regeneration over time, irrespective of age and PMI. Taken together with evidence from grafting or parabiosis between animals of different ages (Carlson & Faulkner, 1989; Conboy et al., 2005), implicates the systemic environment or the local interstitial environment within which they undertake regeneration, rather than the myogenic cells themselves, as the dominant cause of age‐related diminution of regenerative response in skeletal muscle.

The model we present here, although imperfect in some respects, provides the closest experimentally available simulation of human muscle regeneration *in vivo*, notably the excellent reproduction of normal human muscle architecture within these grafts (Figure 7). As an intermediate between mouse and human, xenografts of muscle fragments present a major advantage over grafts of isolated human myogenic cells. Presently, we lack a reliable model for translation of the detailed information being extracted from investigation of murine muscle pathology to comparable processes in human muscle that is required for analysis of the cellular and molecular mechanisms behind muscle regeneration. Firm understanding of such relationships is essential if we are to adjudge the likelihood that gene and cell therapies from simple mouse models would be efficacious in human trials. Our investigation has familarized us with the practical and technical problems to be resolved if we are to fully exploit muscle xenograft models for this purpose. Most important, if we are to minimize inter‐procedure variability that we encountered is the organization of a standardized protocol to supply cadaver muscle from a single dedicated source. The prolonged postmortem survival of myogenic properties within muscle samples provides the great advantage of establishing a routine standard procedure to produce large numbers of grafts from any given muscle sample to minimize noise. This would permit investigation of the fine details of the molecular and cellular components of human muscle regeneration to provide reassurance that principles established in the mouse are applicable to human.

## 4 EXPERIMENTAL PROCEDURES

### 4.1 Study approval and human muscle samples

Through clinical collaborations and approved Material Transfer Agreements (MTA) with the University of Maryland School of Medicine, Georgetown University Medical Center, Howard University Hospital, and Children's National Hospital, we obtained de‐identified human muscle samples harvested during postmortem autopsy procedures and stored in chilled DMEM. Postmortem samples were described only by age, sex, race, and date/time of death and autopsy. Exclusion criteria for human samples included patients with blood‐borne infectious diseases (i.e., HIV, Hepatitis) or neuromuscular diseases. This study was approved by the Institutional Review Board (IRB) of Children's National Hospital and Children's Research Institute.

### 4.2 Xenograft surgical procedure

Animal procedures were approved by the Institutional Animal Care and Use Committee (IACUC) of Children's National Hospital and Children's Research Institute. Female and male NOD. Cg‐*Rag1^tm1Mom^ Il2rg^tm1Wjl^*/SzJ (NRG) immunodeficient mice (Pearson et al., 2008) aged 2–4 months were used for this study (007799, Jackson Laboratories). Donor human muscle was trimmed into approximately 8 × 4 × 2 mm strips. Mice were anesthetized with 2%–5% isoflurane throughout the procedure. The tibialis anterior (TA) and extensor digitorum longus (EDL) muscles were removed from the anterior tibial compartment, and a strip of human muscle was placed in the empty anterior compartment and ligated with 6/0 POLYPRO non‐absorbable sutures (CP Medical) to the tendons of the peroneus longus (Zhang et al., 2014). The skin was then closed with Histoacryl surgical glue (B. Braun) and Reflex stainless steel wound clips (CellPoint Scientific). Buprenorphine SR (1 mg/kg) was administered subcutaneously post‐surgery for pain control.

### 4.3 Histology and immunostaining

Frozen muscles were sectioned at 8 μm thicknesses using a Leica CM1950 cryostat maintained at −20℃. The muscle sections were first stained with hematoxylin and eosin (H/E) for histological assessment of xenograft regeneration. Muscle sections were also immunostained with human‐specific spectrin (SPEC1‐CE, 1:50, Leica) and human‐specific lamin A/C (ab40567, 1:200, Abcam), while total muscle tissue was marked by 4′6‐diamidino‐2‐phenylindole (DAPI) staining (outside human‐marked tissue) or by immunostaining with fluorescently conjugated wheat germ agglutinin (WGA alexafluor‐647, W32466, 1:500, Thermo Fisher Scientific). Specifically, muscle sections were fixed in ice‐cold methanol for 10 minutes, washed in phosphate‐buffered saline (PBS), and blocked in PBS supplemented with 2% goat serum (GTX73249, GeneTex) and mouse‐on‐mouse (M.O.M.) blocking reagent (MKB‐2213, Vector Laboratories). Muscle sections were incubated with primary antibodies overnight at 4℃ and subsequently probed with Alexa Fluor secondary antibodies (Life Technologies). Finally, muscle sections were mounted with Prolong Gold Mounting Media (Life Technologies) with DAPI for nuclear staining.

### 4.4 Muscle fiber analyses

Human regenerated skeletal muscle fibers were quantified in ImageJ software and defined by human‐specific spectrin membrane staining and human‐specific lamin A/C nuclear staining. Central or peripheral myonucleation was assessed based on the position of myonuclei labeled with human‐specific lamin A/C. Minimal Feret diameter was used to calculate muscle fiber size due to its strength in limiting experimental error that may arise from tissue orientation or the sectioning angle (Briguet et al., 2004).

### 4.5 Microscopy

Images were acquired on the Olympus BX61 VS120‐S5 Virtual Slide Scanning System Microscope with UPlanSApo 20x/0.75 and 40x/0.95 objectives, Olympus XM10 monochrome camera, and Olympus VS‐ASW FL 2.7 imaging software. Analysis was performed using Olympus CellSens 1.13 and FIJI ImageJ software.

### 4.6 Gene expression analysis

Human‐specific myogenic mRNA transcript levels were quantified using TaqMan assays specific for each mRNA target. Human‐specific Taqman probes used include the following: *PAX7*, Hs00242962_m1; *MYOD*, Hs00159528_m1; *MYOG*, Hs01072232_m1; *MYH3*, Hs01074199_g1; *HPRT*, Hs02800695_m1; *18S*, Hs03003631_g1 (Thermo Fisher). Total mRNA was converted to cDNA using Random Hexamers and High Capacity cDNA Reverse Transcription Kit (Thermo Fisher). mRNA transcripts were quantified by qRT‐PCR using the listed TaqMan assays and Taqman Fast Advanced MasterMix (Thermo Fisher) on an ABI QuantStudio 7 real‐time PCR machine (Applied Biosystems). A threshold of Ct > 35 was used to eliminate false positives, while samples which were “not determined” by qRT‐PCR analysis were given a Ct value of 40 (upper limit of detection). Raw data (Ct values) normalized to human‐specific house keeping gene, HRPT, and reported as ΔCt and normalized fold change using the formula, 2^−(ΔΔ^ *^C^* ^t)^. Human‐specificity of TaqMan probes was assessed for mRNA isolated from mouse muscle tissue (negative control).

### 4.7 Statistical analyses

Statistical analyses were performed using the following: one‐way ANOVA with Tukey post hoc comparison and two‐tailed, non‐parametric, and Mann–Whitney test. The precise statistical test employed varied depending on the nature of the analysis, and is listed in the figure legends. Data normality was assessed for all statistical comparisons. All *p* values <0.05 were considered significant. *p < 0.05, **p < 0.01, ***p < 0.001, and ****p < 0.0001. Data represented as scatter plots with the mean ± SD.

## CONFLICT OF INTEREST

The authors declare no competing or financial interests.

## AUTHOR CONTRIBUTIONS

TAP, EPH, and KRW conceptualized the study. JSN, DAGM, MN, and TAP performed xenograft procedures in accordance with Institutional IRB and IACUC approvals acquired by MN, JSN, and TAP. OBI, BTH, MNFL, CTR, and DAH together with their respective clinical pathology departments provided human muscle samples. JSN, DAGM, MN, RH, PU, NFH, and TD performed tissue processing, histology, immunostaining, and microscopy. RH and JSN performed gene expression analyses. JSN, DAGM, and TAP involved in manuscript writing. Final mauscript was reviewed by all contributing authors. JSN and DAGM shared the co‐authorship on this manuscript, having contributed significantly to the execution, analysis and publication of this study. The order of first authorship was arranged according to contributions made to study development and execution, data analysis, and publication of the results. JSN and TAP shared the co‐corresponding authorship on this manuscript.

## Supporting information

Figure S1‐S4

## Data Availability

The authors declare that the main data supporting the findings of this study are available within the article and its Supplementary Information files. Additional data are available from the corresponding author upon request.
