# Supplementary material for: Human muscle stem cells are refractory to aging
Source: Aging Cell. 2021 Jun 5;20(7):e13411. doi: 10.1111/acel.13411 (PMC8282247; doi:10.1111/acel.13411)
Supplement: Supplementary file 1 — Figure S1‐S4 [file ACEL-20-e13411-s001.pdf]

Supplementary Material.

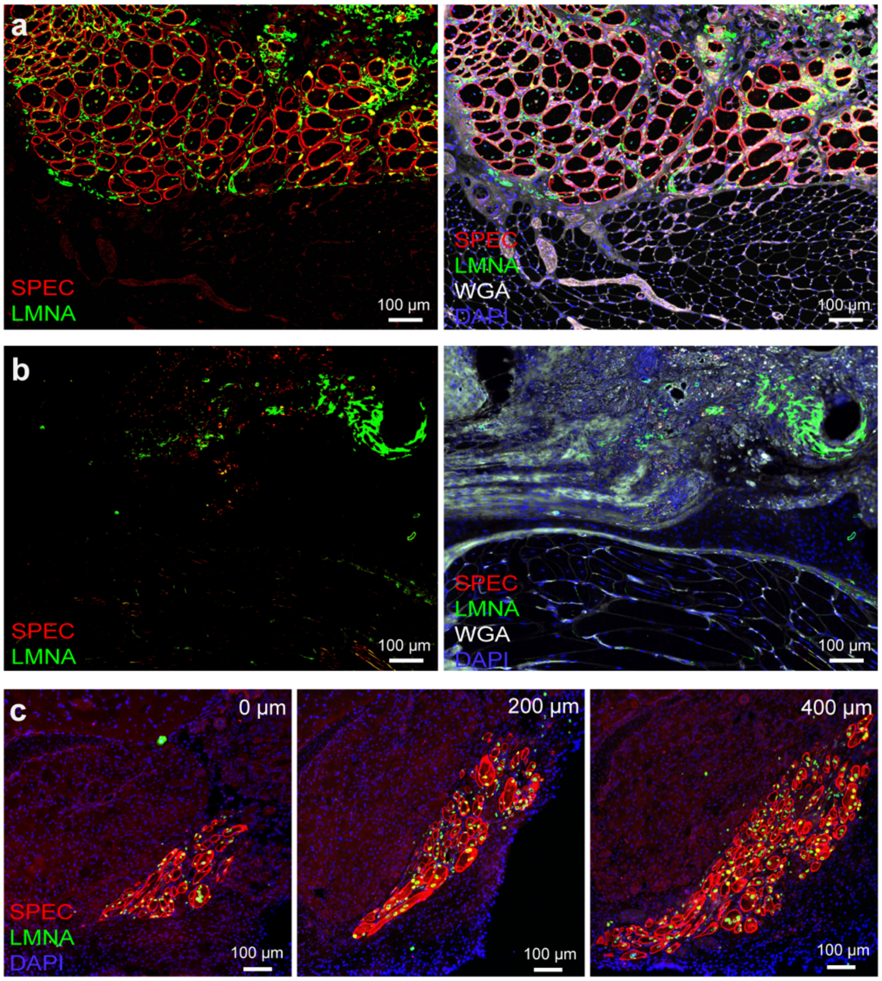

Supplementary Figure 1. Xenograft model to assess myogenic capacity of human skeletal muscle stem cells. a, b) Human skeletal muscle regeneration following muscle tissue engraftment of postmortem skeletal muscle samples in immunodeficient NRG mice. Immunostaining for human-specific spectrin (SPEC, red) and lamin A/C (LMNA, green) proteins identify regions regenerated from human-derived MuSCs, while wheat germ agglutinin (WGA, gray) and DAPI (blue) mark the entire muscle tissue section for reference. Fully-regenerated myofibers of human origin stain clearly for both spectrin and lamin A/C (**a**), while muscles with improper human MuSC-mediated regeneration stained for spectrin and lamin A/C within fibrotic connective tissue along the length of the muscle graft (**b**). **c)** Sampling along the length of the grafted muscle tissue indicates variability with regard to the number of human myofibers in a chosen cross-section of the tissue. Distances shown (top right of images) indicate the depth within the grafted muscle when cryosectioning tissue. Scale bars represent 100 μm; (**a-c**).

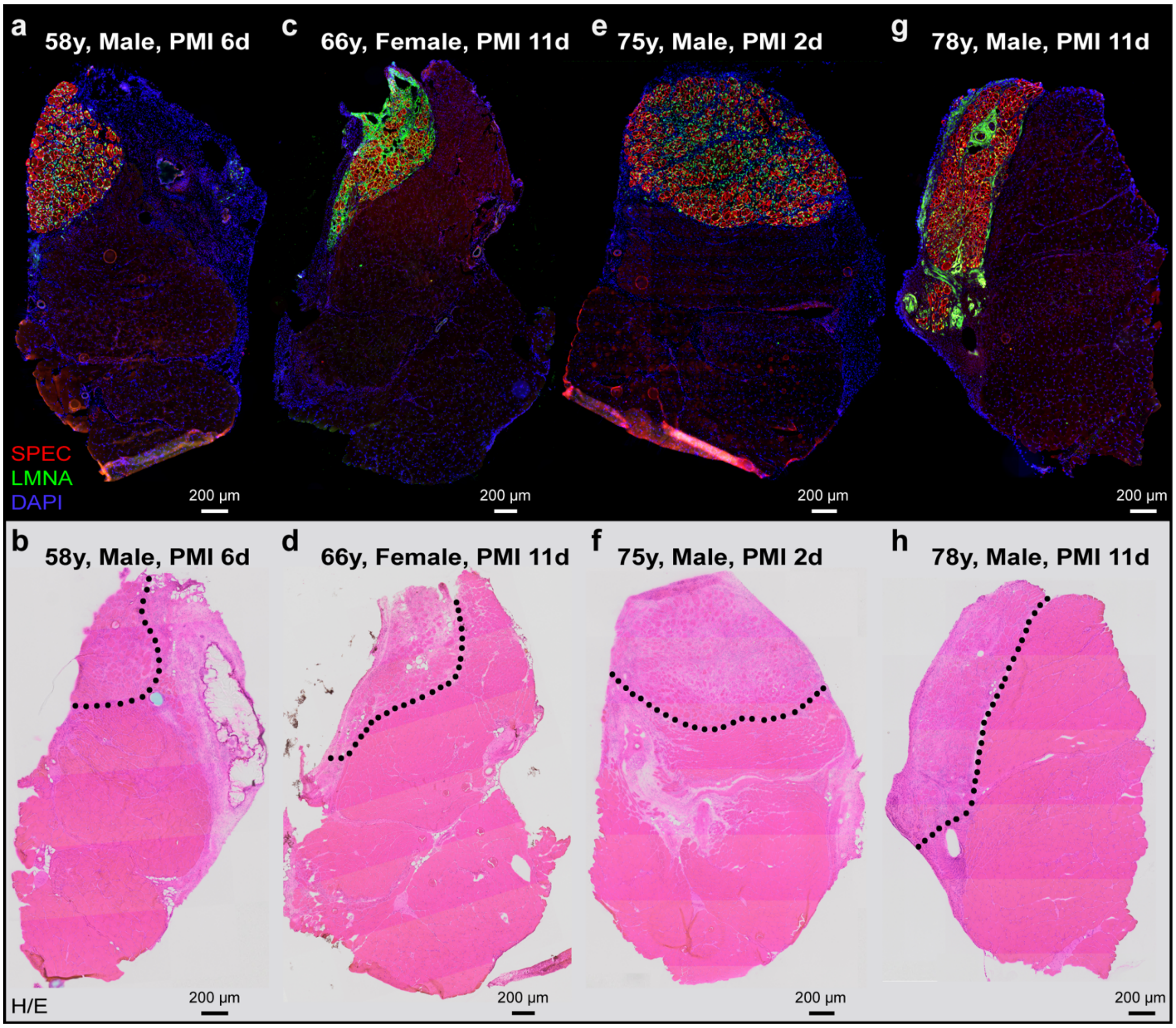

Supplementary Figure 2. Sustained myogenicity of human MuSCs from individuals of advanced age following prolonged postmortem necrosis. a-h) Human skeletal muscle regeneration following muscle tissue engraftment from human cadavers of various ages and postmortem intervals. Immunostaining for human-specific spectrin (SPEC, red) and lamin A/C (LMNA, green) proteins identify regions regenerated from human MuSCs, while DAPI (blue) marks the entire muscle tissue section for reference (**a, c, e, g**). Histology of adjacent serial sections performed by H/E staining shows regrowth of both human and mouse muscle tissue (delineated by black dotted line) harvested from anterior tibial compartment 3 weeks post-engraftment (**b, d, f, h**). Scale bars represent 200 µm (**a-h**). Corresponding magnified imaging shown in **Figure 1**.

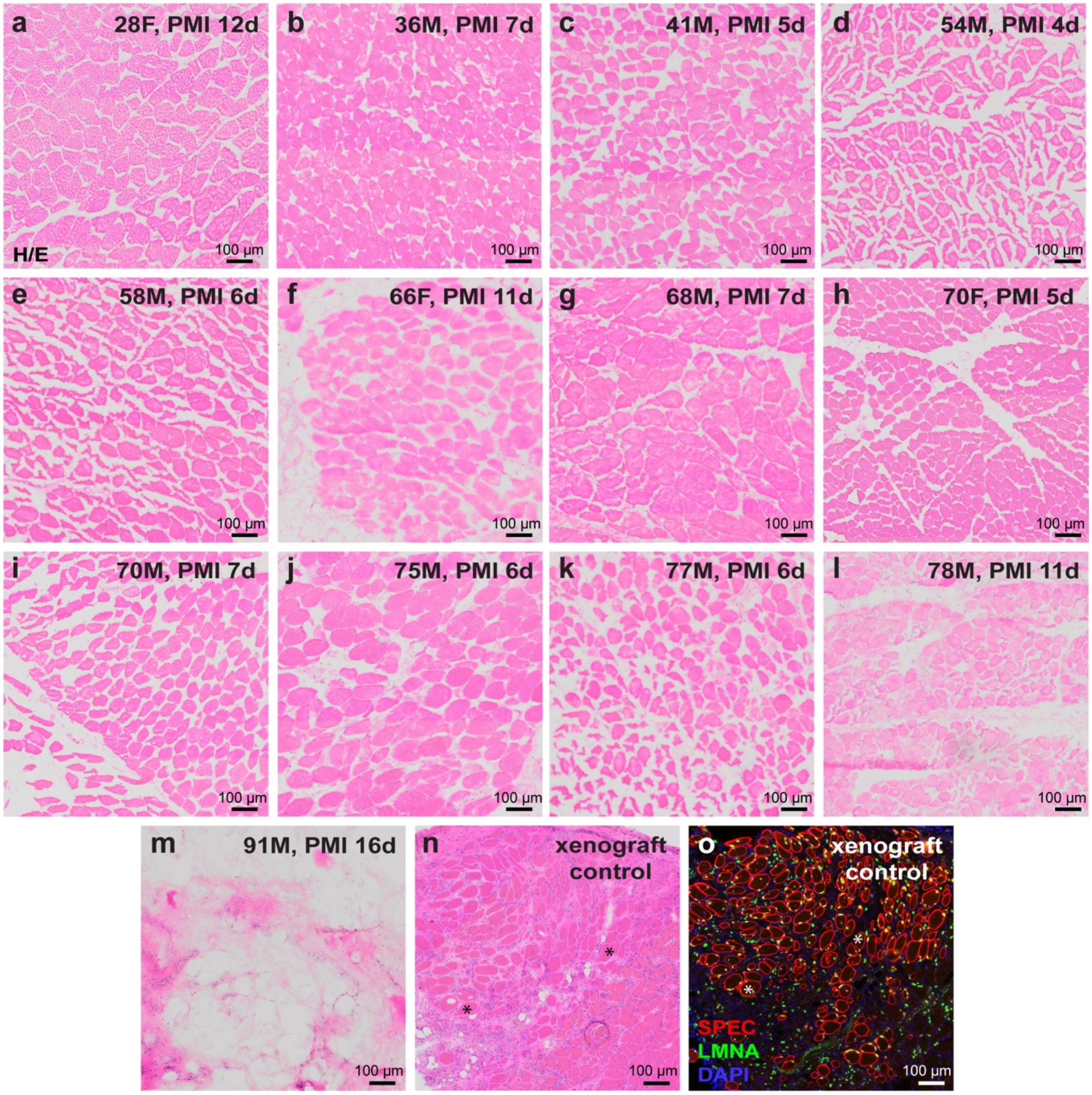

Supplementary Figure 3. Histological examination of human postmortem muscle biopsies. a-m) Histological assessment performed by H/E staining of human postmortem muscle biopsies at the conclusion of each respective xenograft procedure, as denoted in each panel (PMI, d), demonstrating the increasing loss of muscle with increasingly postmortem interval. Despite compromised muscle structure with postmortem interval, our data demonstrates the selective survival of myogenic cells postmortem that undergo successful regeneration upon engraftment in our xenograft model (**Figures 1-3**). **n-o)** Positive control for H/E staining (**n**) of human fibers in a robustly regenerated xenograft sample with corresponding human-specific spectrin immunostaining on a serial cut section (**o**). Asterisks (*) denote tissue orientation between serial sections. Scale bars represent 100 μm (**a-o**).

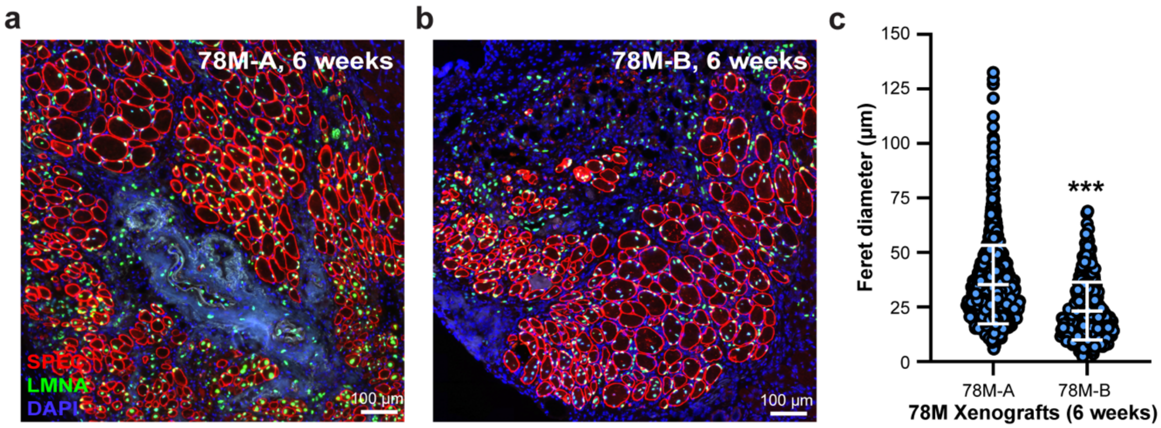

Supplementary Figure 4. Investigation of fiber size distribution in 78-year-old xenograft cohort after 6 weeks of growth. a-b) Increased fiber size observed by immunostaining of human-specific spectrin+ fibers in 78-year-old xenograft cohort 6 weeks post-transplantation. We observed spatial clustering of large diameter fibers, that are largely segregated from clusters of small-diameter fibers within the regenerated region of human tissue. We interpret this finding as a probable effect of innervation by motor nerves penetrating the graft by 6 weeks post-transplantation. Scale bars represent 100 μm (**a-b**). **c)** Minimal ferret diameter quantified from two samples from our 78-year-old, 6-week cohort shown as scatter-plot with mean and S.D. Data demonstrates increased fiber size in both samples relative to other cohorts, and a significant increase for 78M-A specifically resulting in numerous fibers with diameters above 75 μm. Statistical analyses performed by two-tailed, nonparametric, Mann-Whitney test; ***$P <$ 0.001.
